# High-Vacuum Tribological Behaviors of Wear-Resistant WC/a-C:H Coatings with Strong Adhesion on Zirconia

**DOI:** 10.3390/ma18194560

**Published:** 2025-09-30

**Authors:** Zeqing Li, Liang Li, Honghong Zhang, Shubao Shao, Chongpu Zhai, Lunlin Shang, Guang’an Zhang, Minglong Xu

**Affiliations:** 1 State Key Laboratory for Strength and Vibration of Mechanical Structures, School of Aerospace Engineering, Xi’an Jiaotong University, Xi’an 710049, China; zeqingli@xjtu.edu.cn (Z.L.);; 2 National Key Lab of Aerospace Power System and Plasma Technology, Xi’an Jiaotong University, Xi’an 710115, China; 3 State Key Laboratory of Solid Lubrication, Lanzhou Institute of Chemical Physics, Chinese Academy of Sciences, Lanzhou 730099, China

**Keywords:** wear resistance, tetragonal zirconia, hydrogen-containing amorphous carbon, high-vacuum tribological behaviors, adhesin

## Abstract

To improve the applicability of tetragonal zirconia (TZP) in the high-vacuum friction drive field, a strategy combining Cr ion implantation-modified layer and hydrogen-containing amorphous carbon coating was proposed in this study. The designed coating (WC/a-C:H) consists of a Cr bonding layer, a WC-rich load-bearing layer and an a-C:H target layer. The effects of implantation voltage on the adhesion strength of WC/a-C:H coatings were investigated. The tribological behaviors of WC/a-C:H against TZP and TZP self-mated pairs at various loads in high vacuum were comparatively explored. The results indicated that when the TZP substrate was modified by a Cr ion implantation layer, the WC/a-C:H coating showed obviously better adhesion strength. Therein, at the implantation voltage of 30 kV, the coating exhibited the optimal adhesion of 88 N, which was 112% higher than that of the coating on original TZP. Surprisingly, the WC/a-C:H coating featuring maximum adhesion strength also achieved a high friction coefficient (>0.22) and exceptional wear resistance across a wide load range of 0.5~15 N in high vacuum. Compared with the TZP self-mated wear pairs, the wear rates of both the WC/a-C:H coating and its counterparts decreased by 1~2 orders of magnitude. Unlike the severe abrasive wear and plastic deformation of the TZP self-mated pairs, even at 15 N, the WC/a-C:H coating exhibited mild abrasive wear and adhesive wear mechanisms.

## 1. Introduction

Zirconia not only possesses properties typical of traditional ceramic materials, including high hardness, high temperature resistance, and oxidation resistance, but also exhibits outstanding fracture toughness, thus being referred to as the “ceramic metal” [1,2,3]. Tetragonal zirconia (TZP) self-mated pairs feature a high friction coefficient and excellent wear resistance in air. They strike a favorable balance between high frictional forces and low wear rates, thereby showing promising application potential in high-precision or ultra-high-precision friction drive domains, such as piezoelectric inchworm actuators. However, under the condition of high-vacuum or high-load, the wear resistance of TZP deteriorates significantly [4,5], which severely limits its applications in the aerospace field.

The superior mechanical and tribological properties of zirconia-based materials have drawn the focus of numerous scholars [6,7,8,9]. Research has reported that the tribological performance of zirconia (ZrO_2_) can be effectively improved by modulating the microstructure and optimizing the sintering technique. Yang et al. demonstrated that as the grain size, the loading, and the sliding velocity increase, the wear of ZrO_2_ increased significantly. The dominant wear mechanism of zirconia was identified as brittle fracture [4]. Luo et al. discovered that when the mass fraction of TiO_2_ was 5 wt.%, the Ti-YSZ had the superior wear resistance [10]. The wear mechanisms involved plastic deformation and micro-cracking. Chen et al. comparatively evaluated the dry tribological performance of nanostructured and conventional ZrO_2_ coatings [11]. They found that the nanostructured ZrO_2_ tended to generate smaller wear debris, which effectively reduced wear. Frank K. et al. researched the tribological behaviors of alumina–zirconia composite ceramics under different sintering temperatures. They proposed that optimal wear resistance could be achieved at relatively low sintering temperatures (<1525 °C) [12]. Our previous research indicated that even under a low load conditions in high vacuum, the wear rate of TZP self-mated pairs increases remarkably, being 1~2 orders of magnitude higher than that in the air [5]. Currently, the issue of how to significantly improve the wear resistance of ZrO_2_ under the harsh conditions of high vacuum and high load still remains a challenge.

Coating deposition is one effective approach for tailoring the tribological performance of material surfaces. Amorphous carbon (a-C) coatings are regarded as one of the most promising solid lubricating materials, attributed to their high hardness, low friction, high wear resistance and superior chemical inertness [13,14,15,16,17]. The a-C can be classified into two main categories: hydrogen-containing amorphous carbon (a-C:H) and hydrogen-free amorphous carbon (a-C). In comparison with a-C, a-C:H coatings usually possess superior tribological properties in inert gas and vacuum environments [18,19,20]. Erdemir et al. conducted in-depth investigations into the influence of the H/C ratio of plasma gas source on the tribological behaviors of a-C:H coatings. They successfully fabricated the a-C:H coating with ultra-low friction coefficients and extended service lives in a dry nitrogen atmosphere [21]. However, the service lives of a-C:H coatings in vacuum are usually significantly shorter than that in an inert gas environment. Typically, the hydrogen content in a-C:H coatings need to exceed a certain threshold (about 40 at. %) to achieve excellent lubrication and a relatively long service live in vacuum. These a-C:H coatings with lower hydrogen content tended to fail rapidly in vacuum [22,23,24]. The majority of research on the tribological behaviors of a-C:H coatings has focused on their ultra-low friction characteristics [25,26,27,28]. However, there is a significant shortage of research on a-C:H coatings in the field of friction drive. In this field, the friction pair is required to have a high friction coefficient to improve the driving efficiency. Meanwhile, it should also have excellent wear resistance to guarantee the driving accuracy and service life.

To improve the applicability of tetragonal zirconia (TZP) in the high-vacuum friction drive field, a strategy combining a Cr ion implantation-modified layer and hydrogen-containing amorphous carbon coating was proposed in this study. The designed coating (WC/a-C:H) was prepared by PVD/PECVD hybrid deposition techniques, and it consists of a Cr bonding layer, a WC-rich load-bearing layer, and an a-C:H target layer. The microstructure and mechanical properties of the WC/a-C:H coating were characterized. The effects of implantation voltage on the adhesion strength of WC/a-C:H coating were investigated. The tribological behaviors of WC/a-C:H coating against TZP and TZP self-mated pairs at various loads in high vacuum were comparatively explored. The wear mechanisms of WC/a-C:H coating and TZP in high vacuum were further revealed. The WC/a-C:H coating together with the Cr ion implantation-modified layer proposed in this study offers new opportunities for TZP to apply in the high-vacuum friction drive field.

## 2. Experimental Procedures

### 2.1. Coatings Preparation

Commercial yttria-stabilized tetragonal zirconia polycrystal (TZP, Kezhong Ceramic SCI&TECH Co. Ltd., Dongguan, China) was selected as the substrate with dimensions of 20 × 20 × 1.5 mm^3^. The TZP substrates were initially subjected to coarse and fine grinding. Coarse grinding and subsequent fine grinding were carried out in sequence using #400, #800, #1200 and #2000 grit sandpapers. Subsequently, the finely ground specimens were polished with a polishing flannel cloth and diamond polishing paste. The polishing process was continued until the surface roughness of the specimens reached Ra ≤ 0.1 μm. The roughness was evaluated by an aspheric profiler (Taylor Hobson PGI 3D, Leicester, UK). In accordance with ISO standards, a sampling length of 0.08 mm was selected for evaluation, and repeated measurements were carried out at three randomly chosen different positions. The polished TZP substrate was successively immersed in acetone and ethanol for 15 min of ultrasonic cleaning, respectively, so as to remove the contamination on its surface. Subsequently, Cr ion implantation was carried out on the polished surface of the TZP substrate by an ion implanter (3JYZ-8010 MEVVA, Beijing, China) configured with high-purity Cr target (purity ≥ 99.95%). When the vacuum degree was dropped below 3.0 × 10^−3^ Pa, the Cr ion implantation commences. The implantation dose was set at 2 × 10^17^ ions/cm^2^, and the acceleration voltage ranged from 20~40 kV. The TZP substrate subjected to Cr ion implantation at 20 kV, 30 kV, and 40 kV were designated as TZP-Cr20, TZP-Cr30 and TZP-C40, respectively.

The WC/a-C:H coating was fabricated on the original and Cr ion-treated TZP substrates by a multi-arc magnetron coupling deposition system (Hauzer Flexicoat 850, Venlo, The Netherlands). Prior to the deposition of the coating, the substrates were etched by Ar^+^ for 15 min to further improve the surface quality of the substrate. Subsequently, the Cr bonding layer, the Cr/WC transition and WC load-bearing layers, the WC/a-C transition and a-C:H layers were deposited in sequence. The Cr, Cr/WC and WC layers were prepared using the magnetron sputtering technique with high-purity Cr and WC targets. The a-C:H layer was prepared by plasma-enhanced chemical vapor deposition (PECVD), with acetylene as the gas source at a flow rate of 280 sccm. The main preparation parameters of the coatings were shown in Table 1.

### 2.2. Characterization Methods

The surface microstructures of the TZP substrates and WC/a-C:H coatings were characterized by a field-emission scanning electron microscope (FESEM, Zeiss Gemini 500, Baden-Württemberg, Germany). The cross-sectional microstructures and elemental distribution were further observed by a transmission electron microscope (TEM, JEM—ARM300F2, JEOL, Tokyo Metropolis, Japan) equipped with energy disperse spectrometer (EDS). The cross-sectional specimens of the coating with a thickness of less than 100 nm were prepared by a focused ion beam system (FIB, FEI-Helios Nanolab 600i, Hillsboro, OR, USA).

The phase composition of the TZP substrate was detected using an X-ray diffractometer (XRD, Bruker D8 Advance, Karlsruhe, Germany) with Cu-Kɑ radiation. The testing parameters were set as the scanning range from 20° to 90°, the step size of 0.02° and the scanning time per step of 0.3 s. The Raman spectra of the WC/a-C:H coatings from 800 cm^−1^ to 2000 cm^−1^ were measured by a laser Raman spectroscopy (HORIBA-LabRAM HR Evolution, Kyoto, Japan) with a 532 nm wavelength laser. The Raman spectrum of the coating was fitted using the Gaussian function to resolve the disordered (D) peak at ~1360 cm^−1^ and graphite (G) peak at ~1580 cm^−1^. The relative contents of sp^2^ and sp^3^ bonds in the coating were semi-quantitatively evaluated by the intensity ratio of the D peak to G peak.

The adhesion strength of the WC/a-C coating and the TZP substrate was evaluated using a scratch testing system (Anton Paar RST300, Aargau, Switzerland). The normal load was increased from 1 N to 100 N with a loading rate of 0.66 N/s. The scratch length was set to 10 mm with the scratch speed at 4 mm/min. The mechanical properties of the TZP substrate and coating were measured by a nanoindentation apparatus equipped with a Berkovich indenter (KLA iNano, Milpitas, CA, USA). The testing was carried out under the load-controlled mode. The maximum load was set at 8 mN, the loading rate was 0.5 mN/s, and the dwell time was 10 s. The maximum indentation depth was maintained at less than one-tenth of the coating thickness to mitigate the influence of the substrate on the measurement results. For each sample, fifteen positions were randomly chosen for the indentation tests to ensure the reliability of the measurement data.

### 2.3. High-Vacuum Tribology Experiment

The tribological behaviors of WC/a-C:H coatings against TZP balls (Φ 6 mm) at different load 0.5–15 N were conducted in a reciprocating type by a ball-on-disk high-vacuum tribometer (Anton Paar VTRB, Aargau, Switzerland). The TZP balls were supplied by Kezhong Ceramic Co. Ltd. (Dongguan, China), whose surface roughness is lower than 0.05 μm, density is 6.0 g/cm^3^, and hardness is HRA90. The tribological properties of the TZP self-mated pairs were also tested in vacuum as a reference. All tribology experiments started at an air pressure of less than 5 × 10^−4^ Pa and lasted for 9 × 10^4^ cycles. The amplitude of the sliding friction was set to 3 mm and its frequency was set to 8 Hz. The experimental temperature was maintained at 20 ± 3 °C. Each set of tribology experiments was replicated at least three times to acquire reliable experimental data.

The cross-sectional profiles of wear tracks on the coatings were measured by an aspheric profiler. The average wear rates were calculated based on the formula *K* = *V*/(*F* × *S*), where *V* represented the wear volume of the sample (mm^3^), *F* was the normal load (N), and *S* was the total relative sliding distance (m). The FESEM were used to observe the wear morphologies of the WC/a-C:H coatings and the TZP counterparts, respectively, and to analyze their wear behaviors. Furthermore, the Raman spectra of the wear scars on the TZP counterparts were detected by the Raman spectroscopy to investigate their tribology behaviors in the high vacuum.

## 3. Results and Discussions

### 3.1. Microstructure

**Figure 1:**
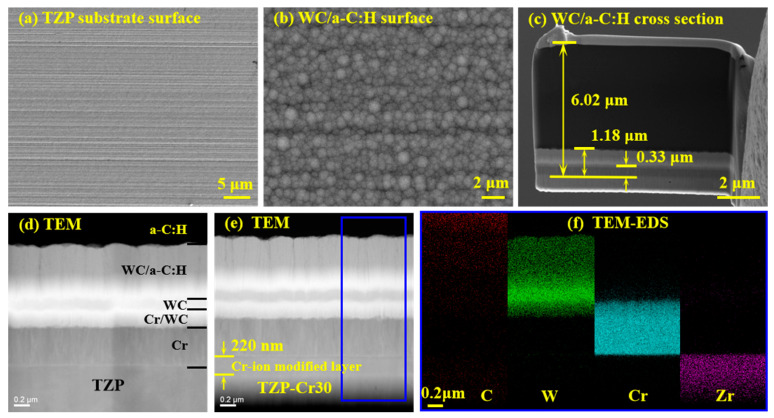
Surface morphology of the TZP substrate (**a**) and the WC/a-C:H coating (**b**), cross-sectional microstructures of the WC/a-C:H coating (**c**) and the interface areas between the coating-substrate of TZP (**d**) and TZP-Cr30 (**e**), the EDS mapping of the marked area of TZP-Cr30 (**f**).

As shown in Figure 1a, the surface morphology of the TZP substrate exhibits distinct mechanical polishing traces. There are numerous parallel micro-scratches, but no obvious pores or crack defects can be observed. Figure 1b presents the surface morphology of the WC/a-C:H coating deposited on the original TZP substrate. The grinding and polishing scratches on the coating surface are not prominent. Instead, it displays as a uniform particle accumulation, and the surface is relatively dense without evident porosity or crack defects. Figure 1c shows that its total thickness is approximately 6.02 μm, and the thickness of the Cr bonding layer is about 0.33 μm. A further observation of the microstructures in the coating-substrate interface regions of the original TZP and TZP-Cr30 substrates was carried out, as shown in Figure 1d,e, respectively. It is revealed that the coating on the surface of the TZP substrate consists successively of the Cr bonding layer, the Cr/WC and WC load-bearing layer, the WC/a-C:H transition layer and the a-C:H layer. In contrast to the original TZP, the TZP-Cr30 exhibits a prominent Cr-ion modified layer near its surface with a thickness of about 220 nm. Furthermore, EDS mapping was conducted on the area marked by the box in Figure 1e. The distribution of C, Cr, W, and Zr elements in the area is presented in Figure 1f. It is clearly revealed that the main elements of each layer of the WC/a-C:H coating. However, the Cr element in the Cr-ion modified layer near the surface of TZP-Cr30 cannot be detected. This is likely primarily attributed to the fact that the dosage of Cr-ion implantation is too low in comparison to that of the TZP substrate.

**Figure 2:**
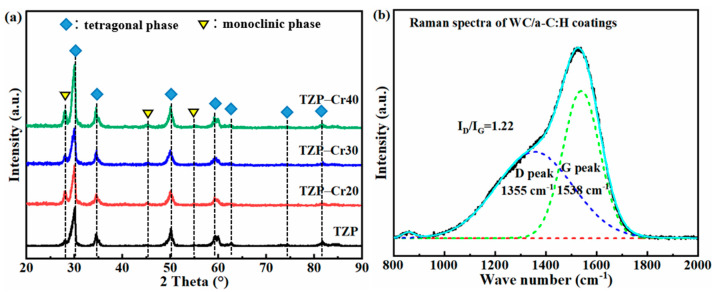
The XRD patterns and phase compositions of original TZP and the TZP-Cr implanted with Cr ion at different acceleration voltage (**a**); the Raman spectra of the WC/a-C:H coating (**b**).

Figure 2a shows the XRD diffraction patterns of the original TZP and the TZP-Cr substrates implanted with Cr-ion under different acceleration voltages. The four TZP substrates exhibit similar diffraction peaks. The peaks at 28.2°, 45.5° and 55.2° correspond to the (−111), (−202) and (122) crystal planes of monoclinic zirconia (m-ZrO_2_), respectively. Additionally, the diffraction peaks at 30.2°, 34.7°, 50.2°, 59.4°, 74.2° and 81.7° are attributed to the (101), (001), (112), (103), (220) and (213) crystal planes of tetragonal zirconia (t-ZrO_2_), respectively. After the Cr ion implantation, the full width at half maximum (FWHM) of the diffraction peaks corresponding to the m-ZrO_2_ phase of the TZP substrate decreases. This indicates that Cr ion implantation induces the transformation of the metastable t-ZrO_2_ phase on the surface into m-ZrO_2_ phase. According to the Toraya formula [29], the volume fractions of m-ZrO_2_ in the near-surface of substrates were further quantitatively evaluated. The results showed that the volume fractions were approximately 20% (TZP), 37% (TZP-Cr20), 30% (TZP-Cr30), and 29% (for TZP-40). It has been confirmed that the transformation from m-ZrO_2_ to t-ZrO_2_ is accompanied by a volume expansion of about 4–5% [30,31,32]. Therefore, the surface of the TZP substrate implanted with Cr ions is likely to have a higher surface compressive stress.

As depicted in Figure 2b, the Raman spectra of the WC/a-C:H exhibits a typical asymmetric broad peak of a-C within the wavenumber range of 1000–1800 cm^−1^. The spectra were fitted using a Gaussian function to obtain a disordered peak (D peak) at 1355 cm^−1^ and a graphite peak (G peak) at 1538 cm^−1^. The intensity ratio of the D peak to G peak (I_D_/I_G_) is commonly employed to qualitatively assess the relative content of sp^3^-C bonds in a-C materials [33,34,35]. The I_D_/I_G_ of the WC/a-C:H coating is about 1.22. In comparison with the a-C coatings deposited by traditional magnetron sputtering in previous investigations [36,37,38], the I_D_/I_G_ of WC/a-C:H coating demonstrates a lower ratio, indicating a relatively higher content of sp^3^-C bonds.

### 3.2. Mechanical Properties

**Figure 3:**
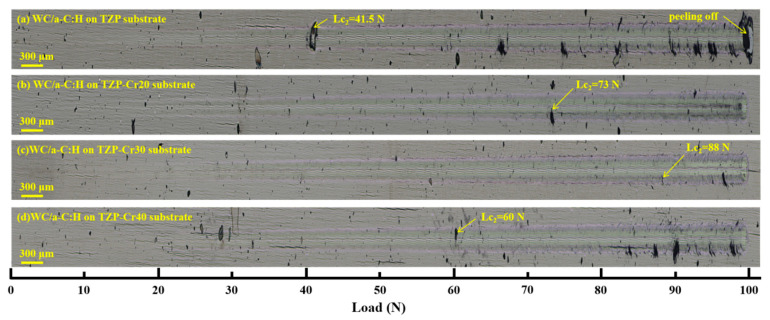
The scratch morphologies of the WC/a-C:H coatings on the different substrates of original TZP (**a**), TZP-Cr20 (**b**), TZP-Cr30 (**c**) and TZP-Cr40 (**d**).

The adhesion of the original TZP and Cr-ion modified TZP substrates with the WC/a-C coating were investigated. Figure 3 shows the scratch morphologies of the coatings on different substrates. With the increase in the normal load, hard coatings generally display distinct damage morphologies [39,40,41]. In this work, the critical load corresponding to the moment when the circumferential cracks first appear in the coating, that is, Lc_2_, is applied to evaluate the adhesion of the coating. This indicates that the adhesion of the WC/a-C:H coatings on the different substrates are approximately 41.5 N (TZP), 73.2 N (TZP-Cr20), 88.0 N (TZP-Cr30), and 60.1 N (TZP-Cr40), respectively. The coating on the original TZP substrate delaminated at a load of 99.8 N, revealing the substrate. However, throughout the entire scratch process, no significant delamination was observed in any of the coatings on the three modified TZP substrates. As the Cr ion implantation voltage increases from 20 kV to 40 kV, the coating adhesion exhibits a trend of first increasing and then decreasing. When the Cr ion implantation voltage is 30 kV, the WC/a-C:H coating on the TZP-Cr30 substrate possesses the highest adhesion, which is about 112% higher than that of the original TZP substrate. This can be ascribed to the fact that the modification of the Cr-ion implantation can reduce the disparity in physical properties between the TZP and the Cr bonding layer enhancing the compatibility of the TZP surface with the bonding layer. The WC/a-C:H coating on the TZP-Cr30 substrate featuring the most optimal adhesion was selected for further investigations into mechanical and tribological performance.

**Figure 4:**
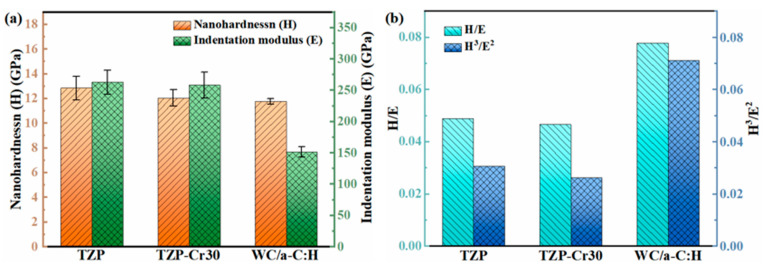
The nanohardness (H), indentation modulus (E) (**a**) and the toughness evaluation parameters H/E and H^3^/E^2^ (**b**) of TZP, TZP-30Cr, and WC/a-C:H coatings.

Figure 4a shows the nanohardness (H) and indentation modulus (E) of the original TZP, the TZP-Cr30 and the WC/a-C:H coating. The TZP substrate and WC/a-C coating have the highest and lowest nanohardness, respectively, with about 12.9 ± 0.9 GPa and 11.8 ± 0.2 GPa. For the TZP-Cr30 substrate subjected to the Cr ion implantation, its nanohardness and indentation modulus decrease slightly to 12.0 ± 0.7 GPa and 258 ± 20 GPa. It may be attributed to the formation of a large number of metallic bonds resulting from the implantation of high-dose Cr ions. Notably, although the nanohardness of the WC/a-C:H coating is comparable to that of the TZP-Cr30 substrate, its elastic modulus is merely 151 ± 10 GPa and ~50% lower than that of the substrate. The parameters of H/E and H^3^/E^2^ were further calculated to evaluate the toughness of the WC/a-C coating and substrates. Many studies have reported that these parameters are usually positively correlated with the toughness of hard materials [42,43]. In comparison with the substrates of TZP and TZP-Cr30, the WC/a-C:H coating demonstrates the evidently highest values of H/E and H^3^/E^2^, indicating that it possesses the optimal toughness. Therefore, depositing the WC/a-C:H coating on the TZP substrate can not only remain a relatively high surface hardness but also significantly improves the toughness of its surface.

### 3.3. High-Vacuum Tribological Behaviors

**Figure 5:**
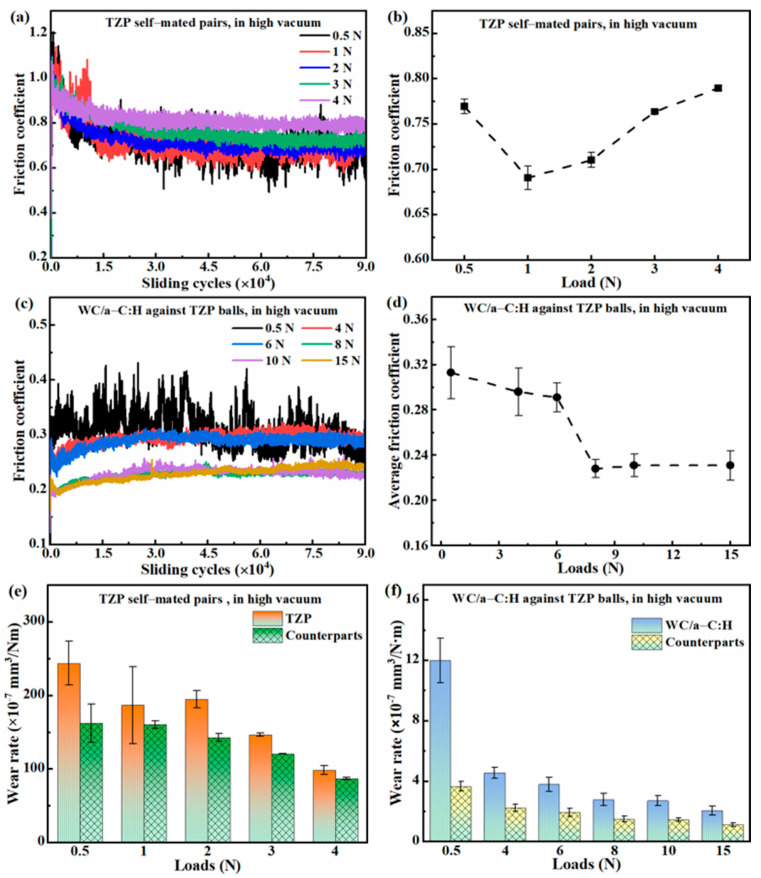
The friction coefficient curves (**a**,**c**), the average friction coefficients (**b**,**d**) and the average wear rates (**e**,**f**) of the TZP self-mated pairs and the WC/a-C:H coatings against TZP balls at different loads in the high vacuum.

Figure 5a displays the friction coefficient of the TZP self-mated pairs as a function of time under loads ranging from 0.5 N to 4 N in high vacuum, hereafter simply referred to as the friction coefficient curves. At the low loads of 0.5–1 N, its friction coefficient curves exhibit continuous and substantial fluctuations within 0.5–0.9. However, when the load is increased to 2.0–4 N, its friction coefficient rapidly reaches a stable phase with relatively minor fluctuations. Figure 5b presents the average friction coefficients of the TZP self-mated pairs during stable phases. It clearly demonstrates that its average friction coefficient gradually increases from 0.69 ± 0.013 at 1 N to 0.79 ± 0.003 at 4 N. At the low load of 0.5 N, its average friction coefficient is relatively high, about 0.77 ± 0.008. Since the maximum measurable frictional force of the tribometer employed was restricted to 10 N, the friction tests of the TZP self-mated pairs at higher loads were not carried out.

Figure 5c shows the friction coefficient curves of the WC/a-C:H coatings against TZP balls at different loads ranging from 0.5 N to 15 N in high vacuum. Similarly to the TZP self-mated pairs, at the low load of 0.5 N, its friction coefficient curves also present obvious fluctuations within 0.25–0.45. In the wide load of 1.0–15 N, its friction curve quickly entered a stable phase and maintained the relatively stable friction coefficient throughout the entire experimental duration. This indicates that the WC/a-C:H coating has excellent adaptability to the wide range of loads in the high vacuum. Interestingly, with the increase in the load, the friction curves of the WC/a-C coatings present a downward trend, which are notably distinct from those of the TZP self-mated pair. As shown in Figure 5d, the average friction coefficients of the WC/a-C:H coatings range from 0.29 to 0.31 at loads 0.5–6 N, while at loads 8.0–15 N, it decreases to approximately 0.23. Based on its friction coefficient, the wide-load can be classified into two stages: a low-load stage ranging from 0.5 N to 6 N and a high-load stage of 8–15 N. Firstly, within each load stage, the friction coefficient curves nearly overlap, suggesting that the friction coefficient is scarcely influenced by load variations. Secondly, in the high-load stage, its average friction coefficient is about 23.3% lower than that in the low-load stage. The stage-dependent variation in the friction coefficient of the WC/a-C:H coating with load might be associated with the evolution of the microstructure at the friction interface at different loads.

Figure 5e,f present the average wear rates of the TZP self-mated pairs and the WC/a-C:H against TZP counterparts at different loads, respectively. As the load increases, the wear rates of both friction pairs display a gradually decreasing trend. The TZP self-mate pairs show the highest and lowest wear rates at loads of 0.5 N and 4 N, about (2.44 ± 0.30) × 10^−5^ mm^3^/Nm and (9.86 ± 0.62) × 10^−6^ mm^3^/Nm, respectively. However, at 0.5 N and 4 N loads, the wear rates of WC/a-C:H coatings are about (1.20 ± 0.15) × 10^−6^ mm^3^/Nm and (4.56 ± 0.37) × 10^−6^ mm^3^/Nm, respectively, which are significantly reduced by 95.2% and 95.4% compared to the corresponding wear rates of TZP. The WC/a-C:H coating has the lowest wear rate of about (2.07 ± 0.31) × 10^−7^ mm^3^/Nm at the high load of 15 N. Moreover, the wear rates of the counterparts against WC/a-C:H coatings at loads of 0.5–15 N range from (1.13–3.66) × 10^−7^ mm^3^/Nm, while those of the counterparts against TZP at 0.5–4 N are in the range of (0.86–1.62) × 10^−5^ mm^3^/Nm. The wear rate of the counterparts against WC/a-C:H is also reduced by one to two orders of magnitude compared with that of the TZP self-mated pairs.

Under the high-vacuum conditions and the wide load ranging from 0.5 N to 15 N, the WC/a-C:H coating not only maintains a relatively high coefficient of friction (>0.22) but also demonstrates excellent wear resistance. This demonstrates its outstanding tribological properties suitable for vacuum friction driven applications. It can be ascribed to two factors. Firstly, the Cr ion implantation modification of the inert zirconia surface, combined with the Cr bonding layer, effectively enhances the adhesion strength between the WC/a-C:H coating and TZP substrate. Secondly, the design of the WC-rich hard ceramic layers is conducive to improving the load-bearing capacity of the a-C:H top layer.

### 3.4. High-Vacuum Wear Mechanisms

**Figure 6:**
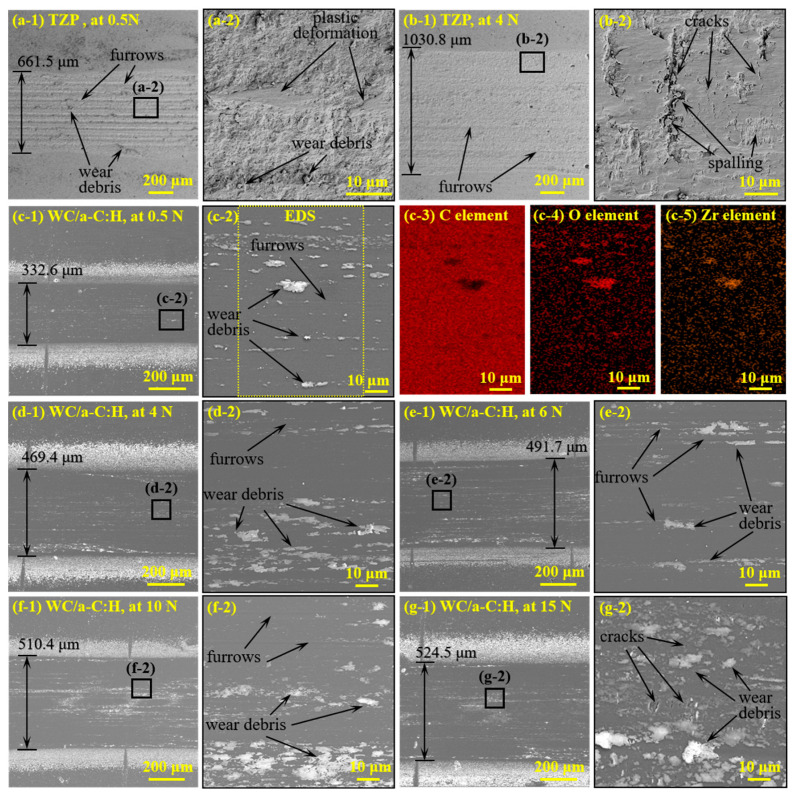
The wear morphologies and EDS analyses of the TZP (**a-1**–**b-2**) and the WC/a-C:H coating (**c-1**–**g-2**) under different loads in the high vacuum environment.

Figure 6(a-1–b-2) shows the surface morphology of the wear tracks of the TZP at loads of 0.5 N and 4 N in high vacuum. It can be clearly seen that even under the low load of 0.5 N, the wear track of TZP is covered with severe furrows and a large amount of wear debris. Figure 6(a-2) presents an enlarged view of the marked region of the wear track, clearly demonstrating obvious local spalling and plastic deformation zones on the worn surface. At 4 N, the width of the wear track increases significantly to about 1030.8 μm. However, its worn surface is smoother, and no severe furrows are observed. Figure 6(b-2) further exhibits the enlarged image of the marked region in the wear track. A substantial number of microcracks, local spallation and severe plastic deformation were observed on its wear track. In the high vacuum, although severe wear took place in the TZP self-mated pairs at both 0.5 N and 4 N loads, the dominant wear mechanisms are different. At the low load of 0.5 N, the wear mechanism of TZP is predominantly severe abrasive wear. In contrast, at the load of 4 N, its wear mechanisms mainly include cracking, spalling and plastic deformation.

In the wide-load of 0.5–15 N, the surface morphologies of the wear tracks on the WC/a-C:H coatings are shown in Figure 6(c-1–g-2). With the increase in load, the width of the wear track extends from 332.6 μm (at 0.5 N) to 524.5 μm (at 15 N), and the wear degree gradually becomes more severe. The worn surfaces of the coating exhibit numerous furrows and wear debris at loads of 0.5–10 N. The EDS analysis of the wear track at 0.5 N, as presented in Figure 6(c-3–c-5), reveals that the white wear debris on the wear track predominantly stems from the TZP counterpart. Figure 6(g-1,g-2) shows the wear track and its local enlarged morphology of the WC/a-C:H coating at the high load of 15 N. It is found that there are some microcracks and local slight spalling on the worn surface.

**Figure 7:**
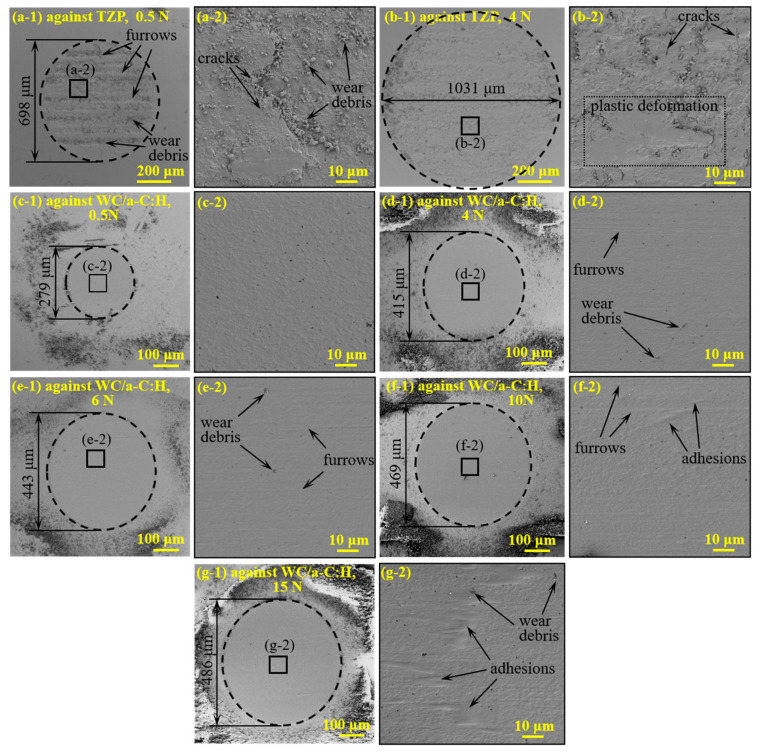
The surface morphologies of the wear scars on counterparts against TZP (**a-1**,**a-2**,**b-1**,**b-2**) and WC/a-C:H coatings (**c-1**–**g-2**) at different loads in high vacuum.

The wear scars of the counterparts against TZP at different loads in the high vacuum are shown in Figure 7(a-1–b-2). Similarly to the wear tracks of TZP, the wear scars on its counterparts also present obvious wear morphologies at loads of 0.5 N and 4 N. At 0.5 N, the surface of the wear scar is rough with numerous severe furrows. When the load was increased to 4 N, the wear scar area of the counterpart expands significantly, reaching a diameter of about 1031 μm. In contrast, at the high load of 4 N, its worn surface is relatively smoother, and the number of furrows is obviously reduced. It can be found that the counterparts have similar wear morphologies to corresponding wear tracks on TZP (Figure 6(a-1–b-2)), suggesting they have same wear mechanisms. With the increase in load, the wear mechanisms of the TZP self-mated pair gradually transitions from distinct abrasive wear to sever plastic deformation, cracking and spalling. It can be attributed to the severe heat accumulation at the friction interface of the TZP self-mated pair at the high load in high vacuum. Previous studies have revealed that in high vacuum environment, even at a low load, the maximum flash-point temperature at the friction interface of TZP friction pairs can exceed 800 °C [5]. Therefore, severe thermal accumulation at high loads can give rise to the degradation of the mechanical properties of the TZP resulting in serious plastic deformation and wear.

Figure 7(c-1–g-2) depicts the wear scars of the counterparts against the WC/a-C:H coatings in the wide-load ranging from 0.5 N to 15 N. With the load increasing from 0.5 N to 15 N, the diameters of the wear scars on the counterparts monotonically increase from 279 μm to 485 μm. Notably, the diameter of its wear scar at 15 N is about 486 μm, which is merely 47.1% of that of the TZP counterpart at 4 N. As shown in Figure 7(c-2–f-2), the worn surface has only slight furrows and a small amount of wear debris, indicating that the wear mechanism of the counterparts against WC/a-C:H coatings is mild abrasive wear. This confirms that in high vacuum, the wear mechanisms of TZP balls against TZP and WC/a-C:H coatings are significantly different. It is worth noting that, in contrast to the smooth wear scar surfaces under 0.5–10 N, Figure 7g-2 clearly reveals the presence of adherents on the wear scar of counterpart at 15 N.

**Figure 8:**
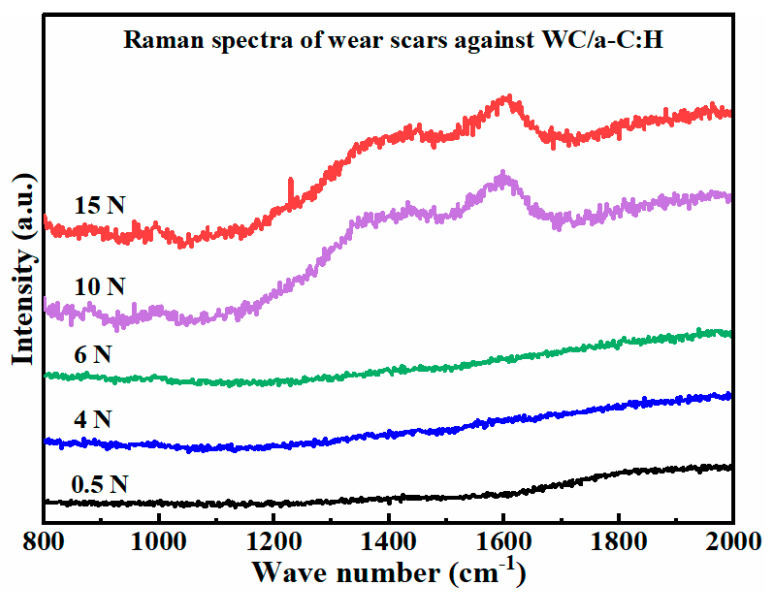
The Raman spectra of the wear scars on the TZP counterparts against WC/a-C:H coatings at different loads.

To further investigate the high-vacuum tribological behaviors of the WC/a-C:H against TZP balls, Raman tests were carried out on the wear scars of counterparts at different loads. Figure 8 shows the Raman spectra of the worn region in the black box in Figure 7(c-1–g-1). At 0.5–6 N, the Raman spectra of the wear scars have no distinct characteristic peaks. However, at the high load of 10–15 N, the Raman spectra of its wear scars display prominent asymmetric double peaks in the range of 1200–1800 cm^−1^, which are the typical characteristic peaks of a-C:H [44,45,46]. It demonstrates that the adherents on the wear scars at 10–15 N in Figure 7(f-2–g-2) are the a-C:H stemming from the WC/a-C:H coating. Evidently, with the increase in the load, obvious adhesive wear starts to occur at the interface between the WC/a-C:H and the TZP counterpart. We reasonably infer that at the high loads ranging from 8 to 15 N, the decrease in the friction coefficient can be ascribed to the formation of the a-C:H transfer materials on the surface of the counterparts. For the WC/a-C:H coating, in the load range of 0.5–6 N, its dominant wear mechanism is mild abrasive wear. By contrast, at the high load of 10–15 N, its wear mechanism primarily involves abrasive wear and adhesive wear.

## 4. Conclusions

To improve the applicability of tetragonal zirconia (TZP) in the high-vacuum friction drive field, a strategy combining a Cr ion implantation-modified layer and hydrogen-containing amorphous carbon coating has been proposed. The designed coating (WC/a-C:H) was prepared by PVD/PECVD hybrid deposition techniques, and it consisted of a Cr bonding layer, a WC-rich load-bearing layer and an a-C:H target layer. The microstructure and mechanical properties of the WC/a-C:H coating were characterized. The effects of implantation voltage on the adhesion strength of WC/a-C:H coating were investigated. The tribological behaviors of the WC/a-C:H coating against TZP and TZP self-mated pairs at various loads in high vacuum were comparatively explored. The main important conclusions of this study are summarized as follows.

Compared with the WC/a-C:H coating on original TZP, the introduction of a Cr ion implantation layer obviously improved the adhesion of WC/a-C:H coatings. At the implantation voltage of 30 kV, the coating exhibited the optimal adhesion of 88 N, which was 112% higher than that of the coating on original TZP.

The WC/a-C:H coating featuring maximum adhesion strength achieved a high friction coefficient (>0.22) and exceptional wear resistance at the wide load range of 0.5~15 N in high vacuum. In comparison with the TZP self-mated wear pairs, the wear rates of both WC/a-C:H coating and its counterparts decreased by 1~2 orders of magnitude.

The TZP self-mated wear pairs displayed different wear mechanisms at various loads in high vacuum conditions. At the load of 0.5 N, the primary wear mechanism was abrasive wear. However, at the load of 4 N, the wear mechanism involved severe plastic deformation, local fracture and abrasive wear.

In the case of WC/a-C:H coating against TZP, within the load range of 0.5 N to 6 N, the dominant wear mechanism was mild abrasive wear. While at higher loads of 10 N to 15 N, the wear mechanisms became more complex, mainly involving abrasive wear and adhesive wear.

## Figures and Tables

**Table 1:** The main deposition parameters of the WC/a-C:H coating.

Deposition Prosess	Cr Target Power/kW	WC Target Power/kW	C_2_H_2_ /sccm	Ar /sccm	Bias Voltage/V	Duration /s
Ar^+^ etching	0	0	0	30	−600	900
Cr	3.5	0	0	150	−70	900
Cr/WC	3.5→0	4.0	0	150	−50	1800
WC	0	4.0	0	150	−50	1800
WC/a-C:H	0	4.0	0→50	150	−50	4200
a-C:H	0	0	280	0	−700	12,600

Note: ‘→’ indicates that this value varies linearly with time during the deposition process.

## Data Availability

The original contributions presented in this study are included in the article. Further inquiries can be directed to the corresponding authors.
